# Heterogeneous approach to nebulization of antimicrobial agents in mechanically ventilated adults in a German tertiary care hospital: a cross-sectional survey

**DOI:** 10.1007/s10096-020-04017-0

**Published:** 2020-09-03

**Authors:** Frieder Pfäfflin, Miriam Stegemann, Norbert Suttorp, Alexander Uhrig, Stephan Achterberg

**Affiliations:** 1 Department for Infectious Diseases and Respiratory Medicine, Charité – Universitätsmedizin Berlin, corporate member of Freie Universität Berlin, Humboldt-Universität zu Berlin, and Berlin Institute of Health, Augustenburger Platz 1, 13353 Berlin, Germany; 2 Antibiotic Stewardship, Charité – Universitätsmedizin Berlin, corporate member of Freie Universität Berlin, Humboldt-Universität zu Berlin, and Berlin Institute of Health, Berlin, Germany

**Keywords:** Nebulization, Invasive ventilation, Antimicrobial, AMS, Heterogeneous, Pneumonia

## Abstract

There is lack of standardization of practices and limited evidence on efficacy and safety of nebulization of antimicrobials. We sought to determine inhalation practices in one tertiary care hospital by performing a cross-sectional survey. Eleven adult ICUs were included in the analysis. Three units followed established protocols. Ventilation circuit filters were exchanged at least daily in all but one units. Dosages of aminoglycosides and CMS depended on indication and unit. Nebulization of antimicrobials was generally regarded as safe and efficacious. Our data indicate that approach to nebulization of antimicrobials may be heterogeneous even in a single center.

**Electronic supplementary material:**

The online version of this article (10.1007/s10096-020-04017-0) contains supplementary material, which is available to authorized users.

## Introduction

Guidelines for nebulization of antimicrobial agents vary. The German guidelines for treatment of nosocomial pneumonia indicate that inhaled administration of antibiotics should be considered in addition to systemic antibiotic therapy in patients with multidrug-resistant organisms (MDRO), which are only susceptible to polymyxins and/or aminoglycosides [1]. Contrarily, the European Society of Clinical Microbiology and Infectious Diseases (ESCMID) does not support the use of nebulization of antibiotics due to a weak level of evidence for their efficacy and the high potential for underestimated risks of adverse events [2]. Furthermore, recent international consensus guidelines for the optimal use of polymyxins advocate the use of adjunctive polymyxin aerosol therapy in patients with extensively resistant gram-negative nosocomial or ventilator-associated pneumonia (VAP) who require intravenous polymyxin [3]. In this journal, Alves et al. reported the results of an international cross-sectional survey on nebulization of antimicrobial agents in mechanically ventilated adults. They found considerable heterogeneity of practice among participating intensive care units (ICU) from different continents [4]. However, little is known about different practices within a single institution. During audits, members of the antimicrobial stewardship (AMS) team of Charité – Universitätsmedizin Berlin had the impression that the approach to nebulization of antimicrobial agents differed among ICUs. We therefore sought to determine inhalation practices systematically in our institution by performing a cross-sectional survey.

## Methods

Charité – Universitätsmedizin Berlin is a large tertiary care hospital with more than 3000 in-patient beds and 25 ICUs on three campuses. As of May 2020, the AMS team comprised four physicians (one microbiologist and three infectious disease physicians) and two clinical pharmacists. Audits with direct feedback are conducted on all wards with high consumption of antimicrobial agents on a regular basis. Within audits, one or two specialist physicians who act as focal persons for AMS represent each ICU. We addressed all 18 adult ICUs. Wards with less than five cases per month were considered ineligible. The survey was built on the SANEME 2 study protocol and adapted to the local situation [5]. Questions focused on key aspects of nebulized antimicrobial agents, which were (1) indications, (2) agents used, (3) dosages, and (4) devices and ventilation settings (see supplementary material). We targeted one specialist physician per ICU who was interviewed by two AMS team members (FP and SA). If the physician was unaware of practices, he was free to ask staff from the unit for assistance. Responses were analyzed by using descriptive statistics.

## Results

Seventeen out of 18 ICUs responded. Six ICUs were excluded as they estimated to treat less than five patients per month with nebulized antimicrobials. The median number of beds of the remaining ICUs was 16 (range 10 to 24). Vibrating mesh nebulizers, ultrasonic nebulizers, and jet nebulizers were used in 6/11 wards (54.5%), 4/11 wards (36.4%), and 1/11 wards (9.1%), respectively. The filter placed on the expiratory limb of ventilation circuits was changed daily in 9/11 wards (81.8%), weekly in 1/11 wards (9.1%), and after every nebulization in 1/11 wards (9.1%). Three wards (27.2%) followed established protocols for administration of nebulized antimicrobials. Bronchodilation before nebulization was conducted always in 5/11 wards (45.5%), as needed in 4/11 wards (36.4%), and never in 2/11 wards (18.2%). Two units (18.2%) increased tidal volume for nebulization of antimicrobials. All other ventilator settings in all of the units remained unchanged. Gentamicin, tobramycin, and colistin methanesulfonate (CMS) were used preferably, while one ward also used liposomal amphotericin B. Prescribed dosages differed depending on indication and ward. Dosages ranged for gentamicin from 40 mg q24h to 80 mg q8h, for tobramycin from 80 mg q24h to 300 mg q12h, and for CMS from 1 MU q24h to 2 MU q8h (Table 1). When nebulized antimicrobials were used for treatment of VAP, they were always combined with systemic antibiotics. Nebulization was regarded as safe and very safe in 5/11 (45.5%) and 6/11 wards (54.5%), respectively. All wards regarded efficacy as good.

**Table 1:** Use of nebulized antimicrobials for specified indications

Drug	Prescribed dosage	No of units administering nebulized antimicrobials for specified indications^a^		
		Prophylaxis of VAT and/or VAP	Treatment of VAT	Treatment of VAP
Liposomal amphotericin B	np			1
CMS	1 MIU q24h	2		
	1 MIU q12h		1	2
	1 MIU q8h	2		
	2 MIU q12h			2
	2 MIU q8h			2
Gentamicin	40 mg q24h	1		1
	80 mg q24h	2		
	80 mg q8h		2	2
Tobramycin	80 mg q24h	5		1
	80 mg q12h	3	1	1
	80 mg q8h		3	5
	80 mg q6-8h		1	1
	300 mg q12h	2		3

*CMS* colistin methanesulfonate, *np* not provided, *No* number, *VAT* ventilator-associated tracheobronchitis, *VAP* ventilator-associated pneumonia

^a^Sum of units may be greater than total number of units as some units use several nebulized antimicrobials for a given indication

## Discussion

Our data indicate that even within one hospital, the approach to nebulization of antimicrobial drugs may be heterogeneous. This may partly be explained by the organizational structure of Charité – Universitätsmedizin Berlin, where ICUs are run by different departments on three different campuses. However, the range in practices may also reflect current national and international guidelines, which give conflicting advice [1–3]. Only three out of eleven ICUs followed established protocols for administration of nebulized antibiotics. Physicians on these wards in particular had extensive experience with inhaled antimicrobials. In one ICU, which is a certified weaning center, inhalation of low dose gentamicin (i.e. 40 mg q24h) is prescribed to all invasively ventilated patients to prevent episodes of VAP. Gentamicin may be replaced by other antibiotics if the colonizing flora is resistant to gentamicin. In case of VAP, other inhaled antibiotics are administered at higher dosage (usually tobramycin 80 mg q8h) and systemic antibiotic treatment is added. Treatment with inhaled gentamicin is resumed after antibiotic therapy for VAP has ended. Physicians of this ward consider this strategy as safe and efficacious; however, further study is necessary to prove or refute its benefit. We found substantial uncertainty regarding nebulization of antimicrobials on most units. In some wards, physicians were unaware of the device used for nebulization and several physicians asked for recommendations on how to approach inhaled antimicrobials. Nonetheless, protocols for filter exchange were in place on all wards and filters were exchanged at least daily in 10 out of 11 units. In the international survey by Alves et al., daily filter changes were carried out in 83 of 216 (38.4%) units [4]. The high percentage of daily filter exchange in our hospital may contribute to the perception of all respondents that nebulization of antibiotics was safe as adverse events in the literature have been linked to obstruction of the expiratory filter [6]. However, respondents in the study by Alves et al. commonly described bronchospasm, cough, and a moderate decrease in oxygen saturation [4]. All these adverse events may occur independently of frequent filter exchanges. Inhalation of antimicrobial drugs was regarded unanimously as efficacious although all respondents admitted that this was merely a subjective estimation. AMS programs face difficulties in giving sound advice on nebulization of antimicrobials. Limited evidence and risk of adverse events caution against liberate administration. On the other hand, there may be a role in treating MDROs and a potential in preventing episodes of VAP. Although the indications for nebulized antibiotics are debatable, technical requirements should be followed to minimize risks of treatment failure and adverse events [7]. The observed heterogeneity of practices in our institution might constitute an argument for institutional-wide standardized procedures and standardized guidelines in general.

## Electronic supplementary material

ESM 1 (PDF 447 kb)
