# Supplementary material for: Heterogeneous approach to nebulization of antimicrobial agents in mechanically ventilated adults in a German tertiary care hospital: a cross-sectional survey
Source: Eur J Clin Microbiol Infect Dis. 2020 Sep 3;40(2):419–21. doi: 10.1007/s10096-020-04017-0 (PMC7471553; doi:10.1007/s10096-020-04017-0)
Supplement: Supplementary file 1 — (PDF 447 kb) [file 10096_2020_4017_MOESM1_ESM.pdf]

1. **Name of the respondent:**
2. **E-Mail of the respondent:**
3. **Name of the unit:**
4. **Specialty of ICU** (e.g. surgical, internal medicine, neurological)
5. **Which device is used for inhalation ?**

ultrasonic nebulizer	jet nebulizer	vibrating-mesh nebulizer	other (please specify)
☐	☐	☐	☐

6. **Is there a protocol available for administration of nebulized anti-infectives ?**

yes	no
☐	☐

7. **Do you make use of bronchodilators before nebulized anti-infectives ?**

always	sometimes	never
☐	☐	☐

8. **How frequently do you change the filter on the expiratory limb ?**

after every nebulization	every day	twice a week	once a week	if necessary
☐	☐	☐	☐	☐

9. **Do you change the respirator setting when administering nebulized anti-infectives ?**

yes	no
☐	☐

10. **If so, which respirator settings are changed ?**

increase PEEP	decrease inspiratory flow	use a constant inspiratory flow	increase inspiratory time	insert an end-inspiratory pause	increase tidal volume	stop the active humidifier	place a filter on the expiratory limb	use sedation to avoid discoordination with the ventilator	use continuous flow or breath actuation
☐	☐	☐	☐	☐	☐	☐	☐	☐	☐

11. **Do you use nebulized anti-infectives for patients with ARDS and NO ventilation ?**

yes	no
☐	☐

12. **Dou you use nebulized anti-infectives for patients with ECMO support ?**

yes	no
☐	☐

13. **Do you make use of intravenous formulations as nebulized anti-infectives ?**

yes	no
☐	☐

14. **For what indication(s) do you prescribe nebulized anti-infective therapy ?**

VAP	VAT	prevention of VAT / VAP	only for VAT / VAP due to MDR (possibly additional systemic therapy)	respiratory tract colonization by MDR	prevention of invasive aspergillosis	treatment of invasive aspergillosis
☐	☐	☐	☐	☐	☐	☐

15. **Which nebulized anti-infectives do you administer for patients with VAT (with dosage) ?**

16. **Do you use systemic anti-infectives as adjunctive therapy in VAT ?**

yes	no
☐	☐

17. **Which nebulized anti-infectives do you administer for patients with VAP (with dosage) ?**

18. **Do you use systemic anti-infectives as adjunctive therapy in VAP ?**

yes	no
☐	☐

19. **Which nebulized anti-infectives are used in prophylaxis of VAT and VAP (with dosage) ?**

20. **Which is your primary objective when administering nebulized anti-infectives ?**

improve survival	prevent adverse events	prevent emergence of resistance	reduce days on mechanical ventilation	treatment of hypoxemia	prophylaxis of respiratory infections
☐	☐	☐	☐	☐	☐

21. **What is the subjectively perceived effectiveness of the nebulized anti-infectives to achieve this objective ?**

very strong	strong	weak	very weak
☐	☐	☐	☐

22. **What is the subjectively perceived safety of nebulized anti-infectives ?**

very safe	safe	unsafe	very unsafe
☐	☐	☐	☐

Abbreviations:

ARDS	acute respiratory distress syndrome
ECMO	extracorporeal membrane oxygenation
ICU	intensive care unit
MDR	multidrug-resistant pathogens
NO	nitric oxide
PEEP	positive end-expiratory pressure
VAP	ventilator-associated pneumonia
VAT	ventilator-associated tracheobronchitis
